# Health Innovation Manchester as AHSS – the Test of a Hypothesis

**DOI:** 10.5334/ijic.5837

**Published:** 2021-08-16

**Authors:** John Rigby, Godwin Chukwukelu, Jose Pineda Mendoza, Jillian Yeow

**Affiliations:** 1 Bibliometrica Ltd, UK; 2 Manchester Institute of Innovation Research, University of Manchester, Manchester, UK; 3 Forctix Ltd, UK; 4 Faculty of Business Administration and Communications, Universidad Privada San Juan Bautista, Lima, PE; 5 Civil Engineering Division L5, Department of Mechanical, Aerospace & Civil Engineering, University of Manchester, Manchester, UK

**Keywords:** AHSS, integrated care, Greater Manchester Health and Social Care Partnership

## Abstract

The ambitious and wide-ranging paper on Academic Health Science Systems [‘AHSS’] [1] proposed a new model for health innovation and stimulated considerable interest. The paper made three main assumptions about AHSS: i) university-based centres should play linchpin roles in health and social care innovation; ii) medical innovation cannot be achieved without links to industry; iii) innovation occurs at the scientific end of a discovery-care continuum. But the paper had a pregnant coda for the NHS, and GM devolution in particular: the authors explicitly linked their view of the need for the integration of university-based research and health care delivery to population level approaches, suggesting that vertically integrated AHSSs should ultimately transform into integrated care organisations. When Manchester’s experiment in the devolution of health and social care as a place-based approach to health and social care began in 2015, Health Innovation Manchester was created as an AHSS to support innovation in the Partnership. Five years after the start of devolution, this short paper, which is based on a longer study of Health Innovation Manchester’s development [2], provides an overdue reflection on the proposition advanced just over a decade ago [1].

## Context and Aim

The authors conducted a study in 2020 of Health Innovation Manchester. The study examined the first five years of Health Innovation Manchester’s history, focussing on the AHSS model upon which Health Innovation Manchester was based, and the wider context of innovation, driven by the integrated care model. Evidence came from documents, board papers, and interviews with senior staff, including members of the executive board past and present.

## Description of the Topic

When devolution of health and social care began in Manchester in 2015, Health Innovation Manchester was created, based on the Academic Health Science System [AHSS] model, to support innovation within the Partnership. The AHSS model stems from organisational innovation in the US. There distinct centres – Academic Health Science Centres (AHSCs) – had emerged, based on integrating at a single location three interdependent activities: research, clinical practice and education. These, so theory went, could be managed to be mutually supporting [3], see also [4]. AHSCs in the US were originally single organisations in one place, but gradually some AHSCs comprised a number of spatially distributed organisations. The AHSC model caught on in the UK, firstly at Imperial College in 2007 [5], then as regionally based centres operating as parts of a larger *federated system*, the Academic Health Science Network (AHSN).

The AHSC model was subsequently broadened in the US to a functionally more diverse AHSS, initially to address the issue of low profitability [6,7]. At the same time, the accountable care organisation/system (ACO) concept in the US health-care reform programme also influenced the AHSC, its emphasis upon connecting actors an important aspect of ‘integration’ [4,5,6,8,9,10]. The wider remit of the AHSS model was held up as better able to exploit academic knowledges, including IP generation. AHSS were exhorted to chase external money, drop topics with little research funding, leverage patient data, work with industry, and innovate in the area of medical training.

In 2010, Dzau, Ackerly et al [1] proposed the AHSS as an innovation model appropriate for the UK. The model was claimed to connect activities on a ‘discovery-care continuum’, along which ideas flow linearly [1] ‘from bench to bedside’, and ‘from local to global’. Their short paper discussed actors and roles – being a theory of the middle range – but did not clarify all the distinctions between innovation and delivery systems, nor the extent of connections between health and social care. Addressing a need for functional integration to achieve outcome-based solutions, the authors, referencing *BusinessDictionary.com*, suggested matrix-management, an approach not always successful [11]. Importantly, the authors reached the conclusion that vertically integrated AHSS’s could evolve ‘to become accountable care organisations … financially responsible for the health of the populations they serve’ [1].

Devolution of health and social care in Manchester was also significantly influenced by the integrated care concept, an approach widely proposed across the UK [12], later NHS England [13] and recently Department of Health and Social Care [14]. The NHS ‘Vanguards’ [15] identify a set number of forms [16], but the conceptual elasticity and quasi-philosophical status can imply ‘anything goes’, albeit with a fixed point being an emphasis upon *outcomes* not structures and processes [17]. As innovation approach, integrated care is abstract concept, more *grand theory* than theory of the *middle range*, neither stipulating roles for specific actors, nor defining ‘translational pathways’ in the manner of the AHSS.

Its proponents also asserted [1] that such systems should ‘morph’ to become integrated care organisations, raising an important question for the Partnership: can and should innovation in health and social care be led academically? A broader question also arises of wider note: if the two models are so closely aligned such that one can become the other, what is the fate of pre-existing integrated care organisations that lack a university medical school, as some do in the UK?

## Discussion and Reflection

At the start of devolution, two models of innovation were ‘in play’ within the Partnership, one based on the AHSS, the other based on integrated care. Establishing the AHSS was first done with essentially a ‘banner-based’ approach to achieving a coherence of plans and resources. When a hoped-for coherence of health research and innovation capabilities showed few signs of materializing, the ‘banner’ approach was dropped. Supported by management consultants, the leadership then proposed a relaunch of the AHSS as an *organization* rather than network or system. Since the 2018 relaunch, the organization has clarified roles and established connections between actors responsible for providing health and social care [delivery] and those responsible for inducing change to provision [innovation]. Governance arrangements have been made to connect innovation capability and awareness of needs through an Innovation Monitoring and Prioritization Committee [‘IPMC’] and the Research and Education Committee [‘REC’], as well as deeper connectivity between all aspects of the GM Health and Social Care Partnership. The IPMC prioritises and oversees the innovation and improvement programmes of some of the work across the GM health and social care system to ensure system-wide engagement before commissioning. The REC includes senior leadership from across GM’s higher education and research infrastructure. While significant outcomes have occurred in terms of improvements to treatment, resulting from the coordination of local and national capabilities and the exploitation of links to industry, and administratively – for example, the sharing of the patient record, achieved in 2021 following action by Central Government [18] – much early work done by the organisation has been organisational and structural, through ‘learning by doing’ focussing on attempting to connect scientific, technological, and research resources through defined use cases.

At the same time that the AHSS model was being explored, the integration model was underpinning service innovation in a range of contexts across the Partnership [19]. Implemented at a range of scales in response to the NHS inspired Vanguards, integration in the Partnership was explored through the Greater Manchester Cancer Vanguard [an Acute Care Collaboration], Salford Together [an Integrated Primary and Acute Care System], and Salford and Wigan Foundation Chain [another Acute Care Collaboration], and Stockport Together [a Multispeciality Community Provider Initiative]. The Partnership itself put two major programmes of work into effect, the Primary Care Strategy, which included a workforce strategy, and the Improving Specialist Care Programme, both major programmes of work significantly changing care and delivery. Within the GM Primary Care Strategy, the AHSS has some limited involvement, in three of the nine areas of the strategy, Digital Enabled Primary Care, Tackling Health inequalities (asthmas) and Improving Quality in Primary care (increasing research), while there was no formal involvement in the Integrated Neighbourhood, Primary and Community Centred Approaches, the Improved Access work, Population Health, or Using information for Improvement and Workforce Development [20].

## Conclusion

The AHSS created by the MOU was only a partially vertically integrated system and while it had high ambitions, it was initially a small-scale initiative with a diverse range of resources that initial optimism of devolution had not aligned coherently. Relaunched as an organisation to solve the problem of coordinating the GM-based scientific and innovation capabilities, Health Innovation Manchester has begun to develop organisational machinery to address the innovation needs of the Partnership. Could then the Partnership’s vision for innovation ever belong solely to an AHSS? At this stage in the running, it seems an unlikely development in the short term, since health and social care innovation driven by integration approaches is currently more far reaching. But there remains value in the concepts of *AHSS* and *integrated care* both influencing practice. Each brings perspective: the former is a theory of the middle range that can be more prescriptive about actors and their roles, while the latter, being a grand theory, supports reflexivity, which is key to innovation. The answer to Dzau’s question is that it may be better to have a balance of perspectives on how to achieve change, and that paradigms may be good, but pragmatism is better.
